# Influence of Water with Oxygen and Ozone Micro-Nano Bubbles on Concrete Physical Properties

**DOI:** 10.3390/ma15227938

**Published:** 2022-11-10

**Authors:** Małgorzata Grzegorczyk-Frańczak, Danuta Barnat-Hunek, Kalina Materak, Grzegorz Łagód

**Affiliations:** 1 Civil Engineering Laboratory, Faculty of Civil Engineering and Architecture, Lublin University of Technology, Nadbystrzycka 40, 20-618 Lublin, Poland; 2 Department of General Construction, Faculty of Civil Engineering and Architecture, Lublin University of Technology, Nadbystrzycka 40, 20-618 Lublin, Poland; 3 Department of Building Materials Physics and Sustainable Design, Faculty of Civil Engineering, Architecture and Environmental Engineering, Lodz University of Technology, Al. Politechniki 6, 93-590 Lodz, Poland; 4 Faculty of Environmental Engineering, Lublin University of Technology, Nadbystrzycka 40B, 20-618 Lublin, Poland

**Keywords:** concrete, water with micro-nano bubbles, frost resistance, mechanical properties, porosimetry, thermal conductivity coefficient

## Abstract

In this study, the possibility of using mixing water containing O_2_ and O_3_ micro-nano bubbles (M-NBs) in concrete technology was investigated. In particular, the effect of micro-nano bubbles on the durability and frost resistance of concrete was analyzed. Concretes with two types of micro-nano bubbles were studied. The physical properties of both the modified concretes and the reference concrete were determined, i.e., specific and apparent density, porosity, weight absorption and coefficient of water absorption. Mechanical parameters based on compressive and flexural strength were tested after 14 and 28 days of curing. Concrete durability was determined on the basis of frost resistance and resistance to salt crystallization. The pore distribution in the cement matrix was determined based on porosimetry studies. The use of water with micro-nano bubbles of O_2_ and O_3_, among others, contributed to a reduction in the water absorption coefficient from 42.7% to 52.3%, in comparison to the reference concrete. The strength characterizing the concrete with O_3_ increased by 61% after 28 days, and the frost resistance after 150 F-T cycles increased by 2.4 times. Resistance to salt crystallization improved by 11% when water with O_3_ was used.

## 1. Introduction

Concrete durability refers to the ability of a concrete structure to resist the aggressive environmental and mechanical actions to which it will be exposed throughout its service life without loss in performance or the need for major maintenance [1]. During use, concrete is exposed to many environmental factors that contribute to its slow degradation and reduced durability [2]. The PN-EN 206-1 [3] and PN-B 06265 [4] standards distinguish seven exposure classes for concrete exposed to environmental conditions: X0—no threat of environmental corrosion, XC—corrosion due to carbonation, XD—corrosion due to chlorides not originating from seawater, XS—corrosion due to chlorides originating from seawater, XF—corrosion due to frost action, XA—corrosion due to chemical aggression and XM—corrosion due to abrasion.

Damage to the structures exposed to harmful environmental conditions has attracted increasing interest from researchers who are trying to mitigate the effects of such damage [5]. To prevent the destruction of concrete caused by salt, carbon dioxide, moisture, frost and aggressive chemicals, it is necessary to improve the durability of the material in adverse environments [6,7,8]. For many years, chemical additives and admixtures have been used in cement composite technology to improve its properties. The most commonly used concrete additives include fly ash, employed as a cement replacement, or micro-aggregate. Fly ash improves the workability of the concrete mix, delays slurry setting, reduces shrinkage and early strength and increases strength in the later stages of concrete maturation [9,10,11,12,13]. Another frequently used additive is silica dust, which seals the concrete structure, reduces permeability to chlorides and sulfates and increases mechanical strength [11,12,13,14,15]. In turn, among the admixtures, one can distinguish liquefying and plasticizing admixtures that decrease the water/cement (w/c) ratio and limit the amount of water in the mixture, as well as aerating admixtures that protect the concrete against the harmful effects of frost, accelerate and retard the setting and enable concrete work to be conducted in periods of reduced/high temperatures [16]. In recent years, additives in the form of nanomaterials have begun to gain great importance in the development of construction. These are the materials consisting of particles with at least one of the three spatial dimensions of less than 100 nm and different physical and chemical properties, compared to their “macro”-scale counterpart [17]. Nanomaterials are classified into [18]: zero-dimensional (nanoparticles); one-dimensional (nanotubes, nanowires); two-dimensional (nanolayers, nanofilms, nanocoatings); three-dimensional (nanocrystalline, i.e., materials containing dispersions of nanoparticles, multi-nanolayer materials, or bundles of nanotubes and nanowires).

Nanomaterials can be manufactured using top-down or bottom-up methods [19]. In the former method, nanomaterials are obtained by disintegrating the starting material into smaller fragments by mechanical force until the desired nanometer size is achieved. This method includes grinding, cutting, shearing, cold rolling, twisting, high pressure, compressive cyclic extrusion and hydrostatic extrusion. In contrast, the bottom-up method involves obtaining nanomaterial from single particles and atoms by chemical synthesis (gas-phase deposition, sol–gel method, electrochemical deposition, precipitation from solutions, chemical reduction, microemulsion method and hydrothermal method).

Nano SiO_2_ is a widely used nanoadditive for concrete. Numerous studies [20,21,22,23,24] show that nanosilica behaves as a nanofiller for the particles of hydrated calcium silicate (Ca-Si-H) in cement, increases the cohesion between aggregate and cement, accelerates the hydration rate of cement, shortens the setting time and enhances early strength, as well as reduces the porosity, absorbability and permeability of concrete, which prevents the potential degradation of the cement composite. A similar effect, especially concerning a decreasing number of pores and cracks, can be achieved by adding nano-eggshell and bagasse [25]. The addition of nano-TiO_2_ to concrete, on the other hand, decomposes organic pollutants as well as contaminants on the concrete surface, giving it self-cleaning and self-disinfecting properties [26]. Nano Al_2_O_3_ increases the compressive strength of concrete, as well as resistance to surface abrasion [18]. Even adding a small number of nanotubes to concrete significantly improves its mechanical properties and prevents cracking [27,28].

Water containing micro-nano gas bubbles is also a type of nanomaterial. It is formed by suspending gas bubbles in a liquid, and due to the unique characteristics of the bubbles (large specific surface area, high stability in the liquid, highly negatively charged zeta potential, high mass transfer efficiency and generation of highly reactive free radicals), it is widely used in numerous industries [29].

For some time, there have been attempts to apply micro-nano bubble water in the production of cementitious composites. It has been demonstrated that replacing tap water with micro-nano air bubble water reduces the weight absorption, capillary absorption of concrete and the penetration depth of pressurized water [30,31,32,33]. Kim et al. [34,35] noted that the application of micro-nano hydrogen bubble water reduced the internal pore diameter and increased the apparent density of the tested materials.

A previous study by the authors [36] showed a 1.9% increase in water absorption by weight for lime–cement mortars containing water with micro-nano ozone bubbles compared to a reference mortar. Micro-nano gas bubble water also improves cement composites in terms of mechanical properties. Taherpour Komishani et al. [37] observed correlations of compressive strength increasing along with the replacement ratio of ordinary water to water with micro-nano air bubbles [31,32,33,34,36,38]. Arefi et al. [31] noticed that the concretes prepared by applying water with micro-nano air bubbles had approximately 19% higher compressive strength, in comparison to the reference concretes. Yahyaei et al. [30] observed an increase of about 3% in tensile strength after 28 days, in comparison to the reference samples. Authors [36] observed an increase in flexural and compressive strengths following 14, 28 and 56 days of curing in lime–cement mortars containing micro-nano bubbles with oxygen, ozone and carbon dioxide.

Most often, chemical air-entraining admixtures (AEA), which reduce the strength of concrete, are used to increase the frost resistance of concrete. The chemical admixture was replaced with completely ecological micro-nano air bubbles, which, unlike AEA, significantly increased the strength of concrete.

## 2. Materials and Methods

### 2.1. Aim and Research Significance of the Experiment

In order to achieve high frost resistance of concrete, concrete mix is aerated using AEA chemical aeration admixtures, the purpose of which is to disrupt capillary networks and take up excess water from freezing capillary pores. Unfortunately, the introduction of additional air into the interior of the concrete inhibits the hydration process and increases the overall porosity of the material, which leads to a decrease in strength. Each additional percentage of air is equal to a decrease in strength of about 5.5% However, there is a lack of any research on the use of nano- and micro-bubbles of oxygen and ozone as an aeration admixture in cement composites. Therefore, the primary objective of the study was fundamental research on the possibility of using micro-nano bubble technology as a new, completely environmentally friendly aerating admixture, which will eliminate a number of problems occurring when using chemical surfactant admixtures in concrete technology, i.e., bubble instability, clusters, agglomerations, uneven distribution of bubbles in the mass of the mixture and negative chemical reactions occurring with other admixtures used in concretes. It seems that limiting the use of chemical aerating admixtures in concrete is most desirable, and the use of non-reactive, environmentally friendly methods of concrete aeration in frost protection should be pursued.

The aim of the paper was to examine and assess the properties of the C20/25 concrete class with micro-nano bubbles of oxygen or ozone admixtures. For the sake of comparison, the same experiments were conducted for the concretes without admixtures. The mechanical properties of concretes as well as the properties corresponding to structure durability under harsh climatic conditions were investigated and an analysis pertaining to their microstructure was carried out. The influence on the durability and frost resistance of concrete exerted by micro-nano bubbles will be analyzed in particular.

### 2.2. Materials

**Figure 1:**
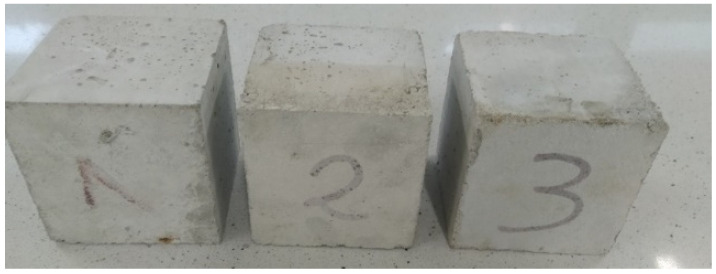
Concretes produced: (1) concrete with micro-nano oxygen bubbles; (2) concrete with micro-nano ozone bubbles; (3) references concrete.

The research was performed to determine the effect of water containing micro-nano bubbles of oxygen and ozone on the physical-mechanical as well as durability properties characterizing concrete. Water containing micro-nano bubbles of oxygen or ozone was substituted for ordinary tap water used to produce concrete. Three types of concrete (Figure 1) with a w/c ratio of 0.35 were made:

- O_2_—concrete containing the water with micro-nano oxygen bubbles;
- O_3_—concrete containing the water with micro-nano ozone bubbles;
- REF—concrete with ordinary tap water.

The composition of concretes was given in Table 1.

Portland cement CEM I 42.5R, manufactured by Cemex Poland, was used as a binder; it was tested according to the EN 197–1 technical standard [39]. The chemical composition and physical properties of the cement are presented in Table 2 and Table 3.

Natural river-derived quartz sand with a 0.0–2.0 mm fraction was employed as fine aggregate. The sand had the following chemical composition: 95.3% SiO_2_, 1.9% Al_2_O_3_, 0.7% Fe_2_O_3_ and 0.35% CaO [42]. The chemical composition of the sand was determined using XRF X-ray fluorescence analysis, allowing quantification of both SiO_2_ and small amounts of other oxides. Elements are detected based on the characteristic wavelength or emission energy of the secondary X-ray radiation. The concentration of a given element is determined by measuring the intensity of its characteristic line. The density of sand was 2.65 g/cm^3^. Gravel with a 2.0–8.0 mm fraction size was employed as coarse aggregate. The density of gravel was 2.64 g/cm^3^. A new-generation carboxylate-based superplasticizer Sika Visco Crete 20 HE, characterized by a density of 1.08 g/cm^3^ and pH value of 4.0, was applied in the amount of 0.5% cement mass (w/c) to reduce the water-to-cement ratio [43]. The liquid hydrophobizing admixture based on an aqueous silane solution was used in the amount of 0.75% in relation to cement weight. The physical properties of this agent were as follows: density 0.98 g∙cm^−3^, viscosity 15 mPa∙s and pH 7; moreover, it was free from volatile organic components (VOCs).

### 2.3. Methods of Obtaining Micro-Nano Gas Bubbles

The micro-nano bubble generator is a system of primary importance for micro-nano bubble production. The generator consists of a membrane of nanoporous material in the form of a tube with irregularly shaped channels through which a mixture of water and gas flows. Ceramic nanoporous membranes and rotary pump technology were employed to perform gas dispersion in water. A tubular SiO_2_ ceramic membrane was applied as a part of the micro-nano bubble generator. The membrane was fixed in a housing made of stainless steel. Compressed gas or air was fed to the generator under pressure, passing through nanopores of the membrane. The generation of micro-nano bubbles consists of two main stages: growth (bubble expansion) as well as detachment from the membrane. During micro-nano bubble generation, a number of forces act on the bubble, which forms via the membrane surface pores. The surface tension holds the bubble inside the nanopore. The resistive force that is exerted by the flowing aqueous phase constitutes a shear force acting on the micro-nano bubble, thus detaching the bubble from the nanopores. Initially, the diameters of the detached micro-nano bubble and pores of the membrane material are the same, creating a hemisphere, and the pressure of the gas in the bubble is maximized. Afterwards, due to micro-nano bubble expansion, the internal gas pressure is reduced. The resistive force resulting from the flowing aqueous phase is enhanced along with the increasing bubble diameter. A bubble is detached from the nanopore when the shear force is higher than the force that holds the micro-nano bubble to the membrane surface. Thus, monodisperse micro-bubbles with a diameter marginally greater than the pore diameter are formed (pore diameter amounts to 50–150 nm).

The described configuration allowed for obtaining typical nanobubble diameters from 80 to >200 nm [36]. The size and quantity of micro-nano bubbles were controlled by such physical parameters as liquid and gas flow rates, as well as pressure. The greater the flow rate of a liquid, the higher the shear stress and the smaller the volume of gas that may detach from membrane surface [44,45,46].

**Figure 2:**
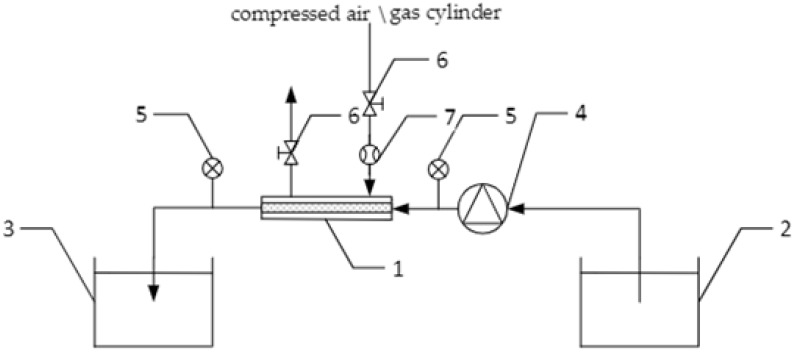
Scheme of the technological installation for the micro-nano bubble water generation: 1—micro-nano bubble generator with a ceramic membrane, 2—primary tank, 3—micro-nano bubble mixing water tank, 4—pump, 5—manometer, 6—valve, 7—rotameter [36].

**Figure 3:**
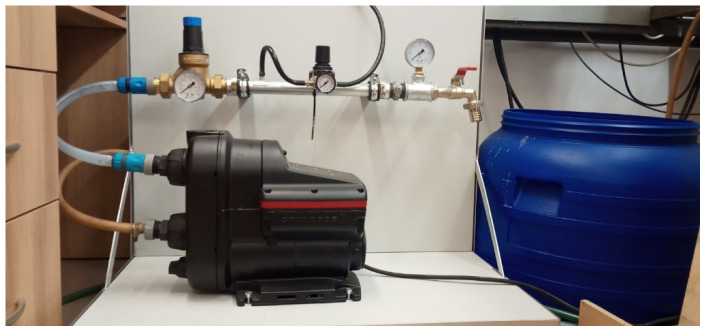
Water generator with micro-nano gas bubbles under laboratory conditions.

An example of a technological system for the generation of nanobubbles in liquid using ceramic membranes is shown in Figure 2. A water generator with micro-nano gas bubbles under laboratory conditions is shown in Figure 3.

The mixing/tap water from the primary tank (2) is pumped using a pump (4) to the micro-nano bubble generator with a ceramic membrane (1). The micro-nano bubble generator is supplied with compressed gas (from a tank). Having passed through the generator, the micro-nano bubble water is directed via a pipe to the tank (3). The installation is equipped with a measurement system, comprising manometers (5) and a rotameter (7).

### 2.4. Methods of Analysis

The concrete samples were prepared in accordance with PN-EN 12390-1:2021-03 [47] and PN-EN 12390-2:2019-07 [48]. Open porosity, total porosity and specific density were determined according to EN 1936:2010 [49]. Open porosity was defined as the ratio of the volume of open pores to the total volume of the sample, expressed as a percentage. In turn, total porosity corresponded to the ratio of all pores to the total sample volume and was determined using the following formula:

P = (100 − S),(1)

where:

P—porosity (%);

S—tightness (%).

(2) $$S=\frac{q_{b}}{q_{r}}\cdot 100$$

where:

q_b_—bulk density, (g/cm^3^);

q_r_—specific density, (g/cm^3^).

The hydrostatic method was employed to determine the bulk density, according to EN 12390-7:2019 [50]. The weight absorbency was determined in line with the PN-88/B-06250 standard [51], while the water absorption coefficient was determined on 100 mm cubic samples according to PN-EN 1925:2001 [52]. The test involved drying the test sample to a constant mass; then, one surface of the sample was immersed in water at a depth of 3 ± 1 mm. Afterwards, the change in its mass as a function of time was measured.

(3) $$C=\frac{m_{i}-m_{d}}{A\cdot \sqrt{t_{t}}},$$

where:

C—capillary water absorption coefficient (g/m^2^·s^0.5^);

m_d_—dry mass of the sample (g);

m_i_—consecutive sample weights during the test (g);

A—water immersed surface (m^2^);

t_t_—the time between the start of the test and the time when the next sample mass is measured m_i_ (s).

The compressive strength was tested following 14 and 28 days of curing in water on cubic specimens with dimensions of 150 × 150 × 150 mm, in line with EN 12390-3:2019 [53]. In turn, flexural strength was determined on the 100 × 100 × 500 mm specimens using the single-point method, via the procedure described in EN 12390-5 [54].

In accordance with the PN-88/B-06250 [51] standard, the ordinary method was used to determine the frost resistance on 12 cubic samples with dimensions of 150 × 150 × 150 mm. Before the test began, 6 samples were saturated with water, weighed and placed in the frost chamber. The samples were frozen by exposure to air at a temperature of (−18 ± 2) °C for a period of 4 h and subsequently thawed by submerging them in water at a temperature of (18 ± 2) °C for a period of 2 h. The 6 reference samples were immersed in water at a temperature of (18 ± 2) °C throughout the freeze–thaw test. After 150 cycles of freezing and thawing (F-T), the samples were removed from the chamber and weighed. Afterwards, a compressive strength test was performed. The result is presented as the average loss in weight of the test specimens in relation to the initial weight and the average decrease in strength of the frozen specimens in relation to the reference specimens expressed as a percentage.

(4) $$\Delta G=\frac{G_{1}-G_{2}}{G_{1}}\cdot 100$$

where:

*G*_1_—average mass of the samples before first freezing, under water saturated conditions (kg);

*G*_2_—average mass of the specimen after its last freezing, under water-saturated conditions (kg).

(5) $$\Delta R=\frac{R_{1}-R_{2}}{R_{1}}\cdot 100,$$

where:

*R*_1_—average compressive strength of comparison samples—unfrozen, water-saturated (MPa);

*R*_2_—average compressive strength of frozen, water-saturated test samples (MPa).

The salt crystallization resistance was determined on 100 mm cubic samples according to EN 12370:2001 [55]. After drying to a constant weight at (105 ± 5) °C, the samples were saturated in a 14-percent sodium sulfate solution, Na_2_SO_4_·10H_2_O, for a period of 2 h at (20 ± 0.5) °C. After the soaking was completed, the samples were dried for 16 h at (105 ± 5) °C. After 15 cycles, the samples were taken out of the dryer and stored for 24 h in water at (23 ± 5) °C. The result is expressed as a percentage, as the relative difference in mass after the test in comparison with the initial dry mass of the sample.

For testing the thermophysical parameters of the tested concretes, a Hot Disk TPS 1500 analyzer (Hot Disk AB) including a probe with Kapton insulation was used.

Mercury intrusion porosimetry (MIP) is a widely used and well-known method to investigate the microstructure of the pore system of porous cementitious materials. The MIP method allows for investigating the structure of pores in the range of 3.5 nm to 500 μm [56]. The mercury intrusion porosimetry assumes that all pores in the investigated material have a cylindrical shape and they are completely able to be filled by a non-wetting liquid, i.e., mercury. The MIP technique also relies on the assumption that mercury can be pressed into pores (of a certain diameter) of solid porous material by applying specific external pressure [57,58]. The information about the mercury intrusion volume and applied external pressure supplies the knowledge and data for the analysis of the microstructure of the material. Knowing the external pressure and using Washburn’s equation [57], the diameter of the cylindrical pore can be calculated [59]:

(6) d =−4 γcosθ/P, 

where:

d—pore diameter (A);

γ—mercury surface tension (g/mL);

θ—contact angle between mercury and solid (°);

P—applied external pressure (psia).

**Figure 4:**
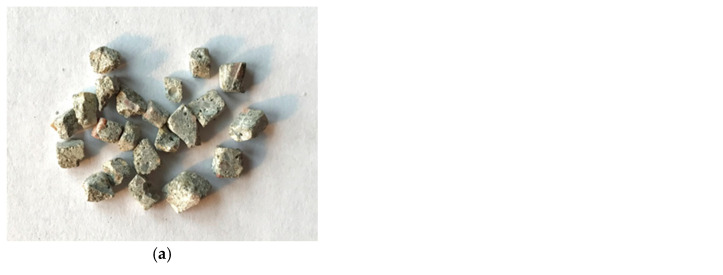

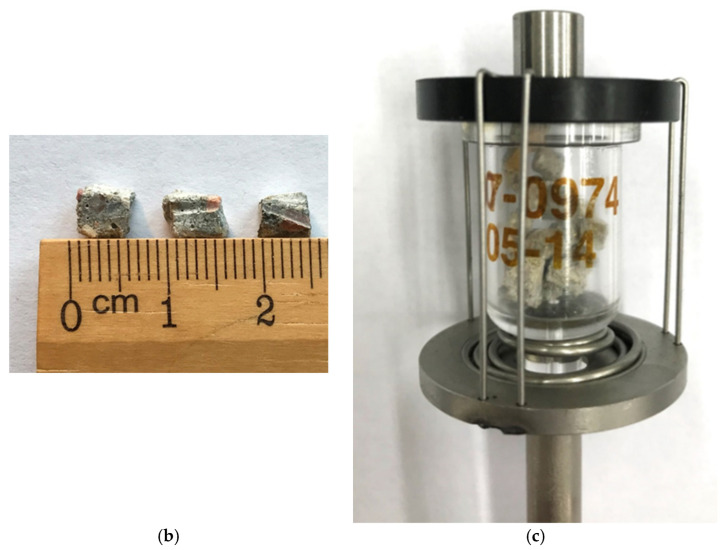
(**a**) Size of the analyzed cement paste chipped off concrete; (**b**) sample of the analyzed cement paste; (**c**) the analyzed specimen in the penetrometer before measurement.

The effect of tap water containing oxygen and ozone micro-nano bubbles on the concrete microstructure was studied using the MIP method. The MIP measurements were carried out on cement paste that was chipped off the original concrete samples. The particle size of chipped pieces of cement paste was around 5 mm (Figure 4a). The specimen of cement paste for the MIP test is shown in Figure 4b. The tests were conducted on two samples of each type of concrete. To remove water from the pores in the material, before the MIP measurement, the samples were dried at a temperature of 50 °C for 14 days. The appearance of the tested sample in the penetrometer before the MIP measurement is shown in Figure 4c. The mass of the tested sample of dried cement paste was around 2–3 g. In the presented research, MIP tests were performed by means of a Micromeritics AutoPore IV9500 device, which can measure pore diameters in the range from about 6 nm to 100 μm. First, the measurement was carried out under low-pressure conditions (with pressure up to 0.54 psia). Subsequently, the sample was placed under high-pressure conditions (with pressure from 0.10 psia up to 60,000 psia). The mercury parameters were as follows: the contact angle was 130 and the surface tension was 458 dyne/cm.

### 2.5. Visualization of the Data via Method of Multidimensional Scaling

The multidimensional scaling (MDS) method was employed to visualize the degree of similarity for the component of the matrix comprising the data related to the characteristics of the tested concretes as well as the differently applied M-NWs of O_2_ and O_3_. The used MDS method [60,61,62] enables the user to obtain a set of points, the distribution of which in a two-dimensional space is in such manner that in the investigated cases (concretes with M-NW O_2_ and O_3_ dosage plus reference), the points that exhibited similarity were located near to one other, whereas dissimilar points were far away [63]. Its purpose was to indicate the general similarity of the analyzed set of concrete samples in a single figure, considering all the measured parameters of the concretes subject to change due to the addition of water with micro-nano bubbles. The scaling was carried out at the beginning with an initial configuration. Afterwards, the points were iteratively shifted, resulting in an improved distance fit to the data until further iterations resulted in no further improvement [64]. Generally speaking, the more accurate the data matching the distances in the MDS, the better the proximity structure is represented by the MDS configuration (e.g., sample measurement set with different modifications). A good MDS fit (e.g., as measured by a value close to zero on the STRESS—Standardized Residual Sum of Squares index) can be verified and interpreted visually, which was performed in this paper.

The morphology and structure of the reference concrete and concrete with MNB were determined using Scanning Electron Microscopy (SEM). The observation was carried out by means of an FEG Quanta 250 microscope (FEI, Hilsboro, OR, USA).

## 3. Results and Discussion

**Figure 5:**
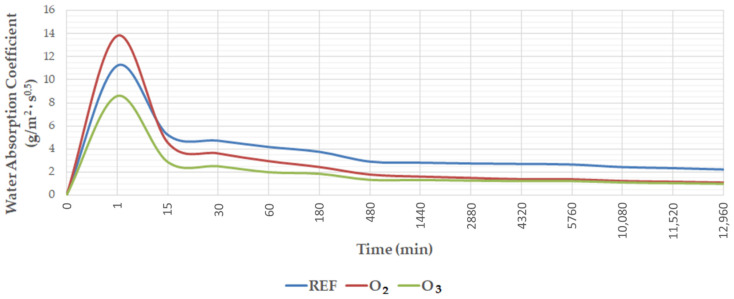
Capillary absorption of concrete as a function of time.

The physical properties of the tested concretes are listed in Table 4, and the water absorption coefficient is presented in Figure 5.

Using the water with micro-nano oxygen bubbles (M-NOBs) instead of ordinary tap water increased the bulk and specific density while decreasing the weight absorption and open porosity characterizing the tested concretes. The water absorption of the concretes with nano-water was found to decrease by about 6.0–7.0%, in comparison to the reference samples. Total porosity decreased by about 6.0–7.0% in all concretes containing micro-nano bubbles, compared to the reference concrete. With the use of micro-nano bubble oxygen–ozone water instead of batch water, the specific density and bulk density of the concretes were slightly enhanced relative to the reference concretes by 1.7% for the M-NOB concretes and 1.3% for the M-NOzB concretes.

Testing showed a decrease in the capillary absorption coefficient in the concretes containing water with micro-nano oxygen and ozone bubbles. The highest water absorption coefficient of 2.812 (g/m^2^·s^0.5^) was observed in the reference concretes, while the concretes containing M-NOzBs and M-NOBs obtained water absorption coefficients 52.3% and 42.7% lower, respectively, than those of the reference concretes.

Yahyaei et al. [30] stated that replacement of tap water with micro-nano air bubble (M-NAB) water decreased the water absorption of test specimens after 30 min by approximately 5% and lowered the pressurized water penetration depth of the test specimens after 28 days of maturation by 8%.

A study by Arefi et al. [31] indicated that the concrete containing M-NAB has a compact and homogeneous structure with few pores. The authors noted that the water absorption tested after 30 min, at 30, 60 and 100% tap water replacement with the water containing M-NAB, decreased by 12, 16 and 20%, respectively, compared to the reference sample. The authors [31] attributed this property to the reduction in capillary pores.

With the decrease in the micro-nano bubble diameter, the negative zeta potential as well as surface tension increase. This contributes to maintaining high stability of the micro-nano bubble in the liquid [34]. Water is pulled by the surface tension into the capillary that is created between the particles. The capillary height increases along with the surface tension. This causes the dispersion of the micro-nano bubble inside the structure of the cement hydration reactant, leading to increased contact of the bubble with more cement particles. Moreover, the micro-nano bubbles move with Brownian motion as well as cause the vibration of the surrounding molecules of water. Due to the high negative potential, water is further dispersed in the cement mixture microstructure. This contributes to the formation of a homogeneous and tight mixture [34].

Kim et al. [34] found that the porosity of cement slurry decreases with an increasing concentration of hydrogen nano bubbles. The average pore diameter also decreased from 38.5 nm to 29.93 nm. The authors explained this phenomenon by the formation of a denser pore structure due to the introduction of fewer bubbles during cement slurry mixing when the concentration of hydrogen nano bubbles was higher. With the change in the distribution of the inner pores, the total porosity gradually decreased. It was found that porosity was greatly affected by a quick decrease in entrained porosity. The ratio of large pores decreased sharply to about 40%, 30% and 15%, while the porosity of the gel slightly increased to approximately 6.8%, 8.8% and 12.3%. The aforementioned pore distribution change indicates the formation of a tight structure in the cement paste. Kim et al. [35] also observed an increase in apparent density over reference samples of 7.6% for lower nano-bubble concentrations and 9.8% for the cement slurry samples made from water containing hydrogen nano bubbles at higher concentrations.

An increase in the density of mortars with water containing M-NB was also observed in a previous study conducted by the authors [36]. Replacing tap water with M-NCD water resulted in a 2.5% increase in specific density and a 1.5% increase in apparent density compared to the reference sample.

Kim et al. [65] observed a decrease in water absorption after repeated immersion of cement mortar in water comprising micro-nano carbon dioxide bubbles (M-NCDBs). According to the authors, this was responsible for the greater carbonation reaction in the water containing M-NCDBs than in the case of carbon dioxide uptake from the atmosphere, which was ultimately responsible for the filling of pores with calcium carbonate. Khoshroo et al. [32] showed a marked effect of the M-NABs-containing water corresponding to reduced capillary absorption of concrete tested following 30 min and 24 h of testing. The authors observed reduced water absorption with an increasing degree of tap water replacement with the M-NABs-containing water (30%, 60% and 100%) by a value of 12%, 16% and 20%, respectively, in relation to the reference samples. SEM studies showed a more homogeneous concrete structure and the presence of smaller pores in comparison to the control concrete. This was confirmed by the study of Mohsen Zadeh et al. [33]. The authors found that capillary absorption decreased due to replacement of tap water with M-NAB water by 16 and 20% at water replacement rates of 50% and 100%, respectively. These conclusions are consistent with previous studies by the authors [36], which observed a decrease in capillary absorption coefficient in the mortars with M-NB compared to reference mortars by 4.7 and 7.9% when oxygen or ozone was applied, respectively.

In a previous study by the authors [36], it was also found that weight absorption increased in the samples with M-NOzBs, in comparison with the reference samples. The study showed a 1.9% increase in weight absorption for the lime–cement mortars with M-NOzB compared to the reference mortar. The study [36] also confirmed a reduced total porosity in the samples containing M-NOzBs and M-NOBs. The lowest porosity was recorded for the mortars containing M-NOBs, which showed a decrease of 13% compared to the reference mortars.

**Figure 6:**
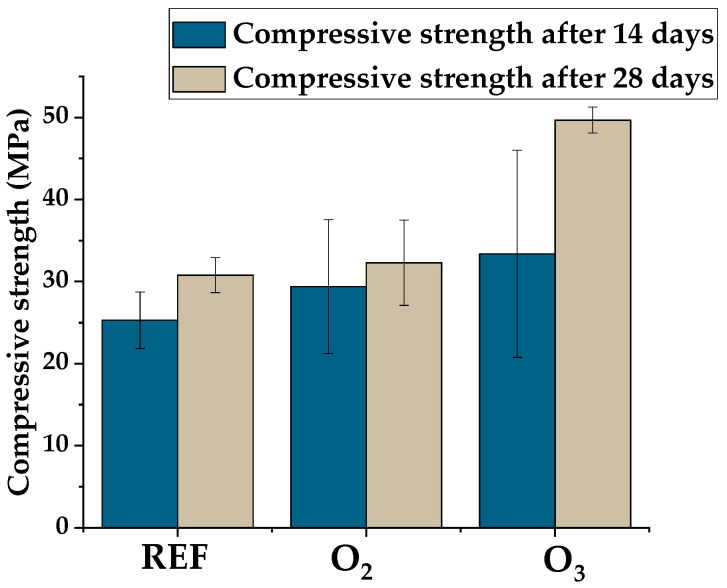
Compressive strength results of the tested concretes.

**Figure 7:**
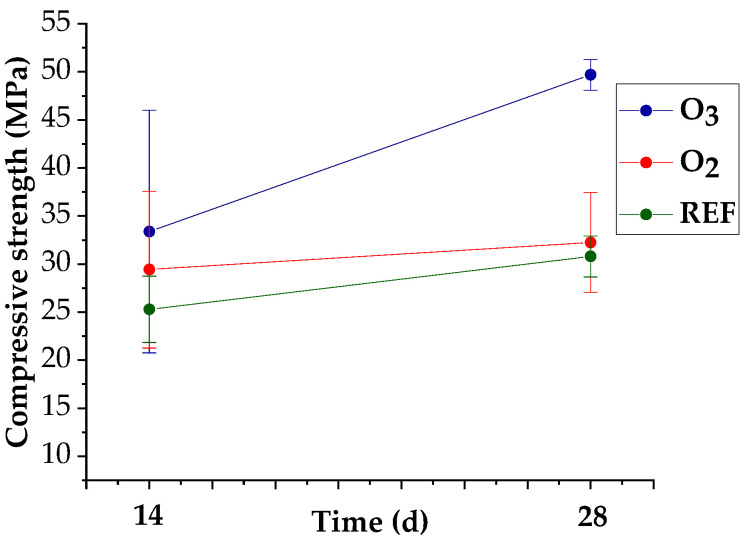
Improvement in compressive strength (%) of concrete.

Table 5 and Figure 6 and Figure 7 show the mechanical properties of the tested concretes. A flexural strength test was performed following 28 days of curing, while a compressive strength test was carried out after curing in water for 14 and 28 days. 

The compressive strength tests indicated that the strength class of the reference concrete was C20/25. The tests showed no effect on flexural strength in the concretes containing the water with M-NOBs and a 5% improvement in flexural strength in the concretes containing the water with M-NOzBs. Replacement of tap water with the water that contained M-NBs of oxygen and ozone improved the compressive strength of the concretes, both after 14 and 28 days of maturation. Improvement in the strength class of the concrete by one class to a value of C20/25 was observed for the MNOB concrete and by four classes for the M-NOzB concretes, which reached a class of C35/45.

Figure 7 shows the compressive strength enhancement between days 14 and 28 of the test.

The concrete with water containing M-NOzBs exhibited a high compressive strength increment between 14 and 28 days of testing. Meanwhile, the reference concrete following 28 days of curing and the concrete with micro-nano oxygen bubbles were characterized by 17.8% and 10% increases, respectively, in compressive strength after the 14th day of testing; an increase in strength of almost 33% was found in the concretes with micro-nano ozone bubbles. This means that in the concretes comprising micro-nano ozone bubbles, a significant increase in compressive strength occurred between the 14th and 28th days of maturation. These concretes also achieved the greatest values of compressive strength following 14 and 28 days of maturation. The strength of the O_3_ concrete increased 1.5 times and that of the O_2_ concrete 1.1 times.

The results obtained confirm the study of Khoshroo et al. [32]. The authors studied concretes prepared with water containing M-NABs with different tap water substitution ratios (30, 60 and 90%). The study showed increases in compressive strength after 28 days of curing of 6, 9 and 13% for the concretes containing 30, 60 and 90% M-NAB water, respectively, compared to the reference concretes. The authors believed that the increase in strength was due to the rapid hydration process of the concrete containing micro-nano bubbles and the high homogenization of the concrete mixture as a result of the floating effect of cement particles with micro-nano bubbles.

Full substitution of tap water with M-NAB water showed an increase in the compressive strength of the concretes in all studies available in the literature [31,32,33,38]. A study by Arefi et al. [31] showed an increase of approximately 19% in compressive strength after 7 and 28 days compared to reference concretes, while Asadollahfardi et al. [38] observed a 13.2% increase in concrete compressive strength after 28 days of curing, compared to a reference sample.

Yahyaei et al. [30] reported an increase in the compressive strength of self-compacting concretes containing M-NAB water at all periods studied. The authors showed that the use of micro-nano bubble water increased the compressive strength of the concretes after 7, 28, 56 and 90 days by about (16, 11.5, 12 and 11)%, respectively, compared to the comparison samples.

A study by Mohsen Zadeh et al. [33] showed an increase of about 14% in compressive strength after 28 days compared to the re-fermented sample, as a result of replacing tap water with M-NAB water. The authors attributed this finding to a higher degree of hydration and better flotation of cement grains.

Micro-nano bubbles increase the hydration of cement particles due to their small size. A reduction in the diameter of the bubble contributes to a higher probability of the micro-nano bubble colliding with the cement particle. Since the micro-nano bubbles reduce the force of water lifting, the flotation coefficient of cement particles is reduced. Therefore, the probability of collision between bubbles and cement particles is much higher than in ordinary tap water [66,67].

In turn, Taherpour Komishani et al. [37] studied the effect of micro-nano bubble water in the production of concrete conditioned in seawater. The authors observed an increase in the compressive strength of concrete made with M-NAB at all tested times, compared to concrete made with tap water. In addition, correlations were observed between the increase in compressive strength and the ratio of tap water to M-NAB water. Complete replacement of tap water with M-NAB water resulted in an (8–9)% increase in compressive strength after 7 as well as 28 days of curing in tap water.

An increase in mechanical properties due to the use of water with micro-nano bubbles was also observed in previous studies by the authors [36]. Replacement of tap water with micro-nano bubble water showed an increase in compressive strength after 14, 28 and 56 days of maturation in lime–cement mortars containing micro-bubbles with oxygen, ozone and carbon dioxide. The largest increase in compressive strength (27.3%) was observed in the samples containing M-NOBs, compared to reference mortars.

Torki et al. [68] studied the effect of M-NAB water on the properties of cement mortars in the presence of polycarboxyl-ether-based super-plasticizer with variable dosage rates. The study showed increases in strength after 7 and 28 days of maturation of 21 and 10%, respectively, in mortars containing M-NABs and superplasticizer at 0.5% by weight of cement compared to the reference samples without M-NABs. Torki et al. [68] observed a large increase in the compressive strength of young-aged concretes compared to reference samples, with the difference decreasing with the age of the concrete. The authors attributed this correlation to the shortened setting time in mortars with M-NABs and the nullification of the negative effect of the superplasticizer on early cement hydration. The mortar with M-NABs reduced the setting time of primary and secondary cement, resulting in faster mortar hardening and faster achievement of higher compressive strength. Hassani et al. came to similar conclusions in their study [66]. The authors observed the highest increase in compressive strength in concretes with w/c = 0.35 by 6, 18.3 and 16.3% after 7, 28 and 90 days of curing, respectively.

In turn, Jebeli et al. [69] observed increases in compressive strength of 23.9, 31.5, 11 and 39.9% after 28 days in the concretes containing M-NABs and fine recycled aggregate in amounts of 10, 20, 30 and 40%, respectively. The authors show that the use of M-NABs can increase the recycling of fine aggregate by up to 40% in concrete, which can protect natural sand resources.

On the other hand, Kim et al. [34] studied the mechanical properties of cement mortars made with water containing hydrogen micro-nano bubbles (HNBs) at different concentrations as a substitute for tap water. The study showed an increase in the compressive strength of the mortars at all tested periods (3, 7, 14 and 28 days of curing). An increase in strength along with the concentration of hydrogen micro-nano bubbles was also observed in the solution. The value of compressive strength in the period from the 3rd to the 28th day of curing was (23.66–35.68) MPa using ordinary hydrogen-bubble water, while the compressive strength of mortars prepared with NHB water was (25.98–37.0) MPa and (28.38–38.67) MPa for water with lower and higher micro-nano bubble concentrations, respectively. Increasing the concentration of NHB water also increased the compressive strength after 28 days of maturation by 3.7% for the water with a lower concentration and by 15.8% for the water with a higher NHB concentration, compared to tap water samples.

In another paper, Kim et al. [35] observed a (1.7–8.6)% increase in flexural strength after just 3 days of curing, depending on the concentration of hydrogen micro-nano bubbles, compared to a reference sample. In contrast, after 28 days, flexural strength had already increased by (2.2–13.5)% with respect to the reference sample.

Yahyaei et al. [30] observed an increase in the tensile strength of self-compacting concretes containing M-NAB water after 28 days by about 3% compared to the control samples. In contrast, a study by Mohsen Zadeh et al. [33] showed increases of (6 and 18)% in the flexural and tensile strengths, respectively, of concrete with M-NABs after 28 days, compared to a reference sample. 

Asadollahfardi et al. [38] observed a 16.4% increase in tensile strength in the concretes with M-NABs, compared to those with ordinary tap water.

A test of the tensile strength of concrete with M-NABs conducted by Taherpour Komishani et al. [37] after just 1 day of curing on cylindrical specimens showed that the specimens conditioned in seawater had 60% higher tensile strength compared to those conditioned in tap water. They also observed a 6% increase in the flexural strength of the concrete with M-NABs after 28 days of conditioning in seawater compared to concrete conditioned in tap water.

The authors’ own research [36] also showed an increase in flexural strength after 14, 28 and 56 days of curing in lime–cement mortars containing micro-nano bubbles with oxygen, ozone and carbon dioxide. The largest increase in flexural strength after 28 days of curing, compared to reference mortars, was observed in the mortars containing M-NCDBs (20.9%).

**Figure 8:**
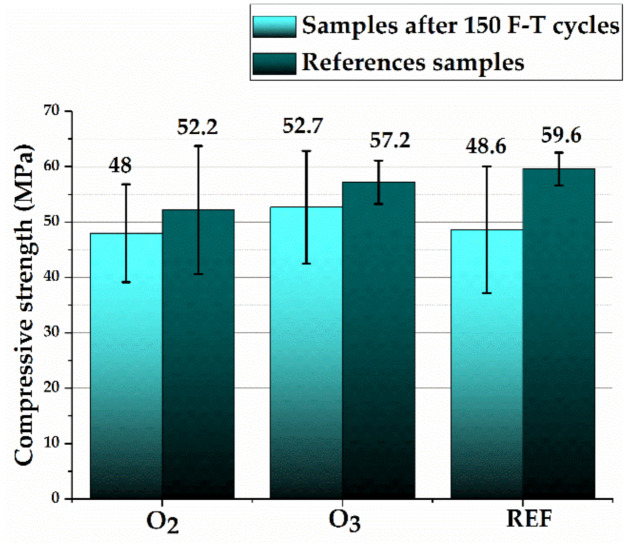
Compressive strength results of frost-treated and comparison specimens.

Table 6 and Figure 8 show the durability properties of the tested concretes. Figure 6 presents the compressive strength values of the samples after 150 freezing and thawing (F-T) cycles as well as the values of strength of reference samples not exposed to frost impact testing. 

All tested concretes were found to be frost-resistant (ΔR ≤ 20% and ΔG ≤ 5%), obtaining an F150 class. The study showed that the concretes containing micro-nano bubbles of oxygen and ozone were characterized by higher frost resistance, expressed as both weight loss and compressive strength decreases, in comparison with the reference samples. The concretes containing M-NOzBs showed the smallest compressive strength reduction following the frost resistance tests, amounting to 7.8%. These concretes also showed the smallest decrease in weight due to frost action and the smallest increase in weight according to the salt crystallization resistance test. The highest mass increase following the abovementioned test was found in the reference samples. These concretes also had the highest open porosity. Frost resistance tests were performed on cubic specimens measuring 10 cm × 10 cm × 10 cm. The results obtained cannot be compared to compressive strength tests performed on 15 cm × 15 cm × 15 cm cubic specimens (Figure 6 and Figure 7). All tested concretes were found to be frost-resistant (ΔR ≤ 20% and ΔG ≤ 5%), obtaining an F150 class. Obtaining lower strength by concretes with O_2_ and O_3_ admixtures is not a negative effect, as the concrete class did not change. The effect of admixtures is clearly visible at the initial stage of maturation and after 28 days. An increase in compressive strength is then observed. However, after 150 F-T cycles, i.e., after 70 days of curing, the increase in strength was somewhat inhibited compared to the reference concrete. The purpose of our study was to demonstrate the effect of M-NOBs and M-NOzBs on the durability of concrete, specifically its frost resistance. Compared to the reference concrete, the decrease in strength after 150 F-T cycles was smaller at 7.8% for M-NOzBs and 8% for M-NOBs. In turn, for the reference concrete, the decrease was as high as 18.5%. Therefore, the effect of the O_2_ and O_3_ admixture can be considered positive. Additionally, the difference in strength between the reference concrete and, for example, that with MNOzBs, was only 2 MPa (i.e., 4%), which is within the error limit, as evidenced by the scatter of results (Figure 8).

**Figure 9:**
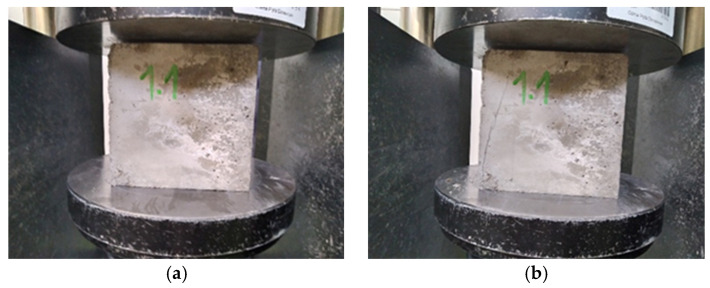
Concrete with micro-nano oxygen bubbles before (**a**) and after compressive strength testing (**b**) following 150 F-T cycles.

**Figure 10:**
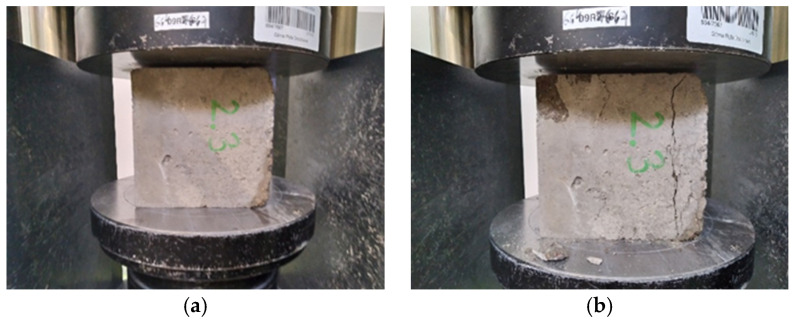
Concrete with micro-nano ozone bubbles before (**a**) and after compressive strength testing (**b**) following 150 F-T cycles.

**Figure 11:**
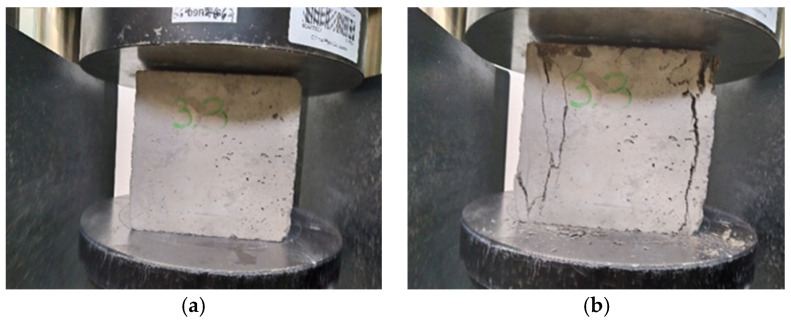
Concrete with ordinary tap water before (**a**) and after compressive strength testing (**b**) following 150 F-T cycles.

Figure 9, Figure 10 and Figure 11 show concretes before and after compressive strength testing following 150 F-T cycles. The photographs of the tested concretes show that the destruction intensity of the samples varied. While the concretes with water containing micro-nano gas bubbles showed a similar failure pattern, the control specimens were characterized by a higher intensity of failure due to the application of a destructive force.

During the literature study, the authors did not find the results of studies testing the durability of cement composites containing water with micro-nano bubbles. On the other hand, it is known from the extensive literature [23,70,71] that its addition in the form of nanomaterial to concrete improves its frost resistance.

Khoshroo et al. [32] observed that replacing part of the cement with metakaolin and using micro-nano bubble water instead of tap water increased the electrical resistance of the concrete. This phenomenon was attributed by the authors to the reduced movement of free ions in the concrete as well as the decrease in the pores of the concrete where water could accumulate. Microstructure studies [32] confirmed the achievement of a denser and homogeneous structure in the concretes containing M-NAB water. The micro-nano bubble water increases the density of the concrete by improving the homogeneity of the mixture, as it intensifies the flotation properties of the cement particles, which consequently increases the contribution of cement particles to the hydration process and contributes to the cement gel formation. On the other hand, accelerating the hydration and cement hardening at an early stage can cause a shorter setting time, flowability of cement mortar reduced workability, in addition to increased durability and compressive strength of cement mortar and concrete [66].

Differences in the microstructure of the concrete made using water with micro-nano bubbles in comparison to the control samples were observed by Hassani et al. [66]. SEM analysis showed a different form of structures in the concretes with M-NABs. On the other hand, microstructure studies showed that the concretes with M-NAB had well-formed crystals with sharp edges and a compact, dense solid form with fine C-S-H gel structures around them. In contrast, thin flakes of calcium hydroxide CH crystals without sharp and thick edges were observed on the control samples. As opposed to the samples with micro-nano bubbles, the crystals present in the control samples were covered with homogeneous C-S-H gel. Microcracks and micropores were also observed on the surface of the control samples. Furthermore, EDX (X-ray spectroscopy) chemical analysis of the concrete showed that the replacement of tap water with the micro-nano bubble water contributed to an increase in the elemental contents of Si, Fe and Al and a decrease in the elemental contents of Ca, Na and Cl compared to the control samples.

A study by Gonzalez et al. [72] on the influence of nanosilica admixture on concrete frost damage showed that the addition of nanosilica at 2% by weight of cement decreased the weight loss after 324 F-T cycles by as much as 47%, compared to the specimens without nano-SiO_2_.

Tarangini et al. [73] observed a (27–31)% decrease in weight loss following 50 F-T cycles in the concretes containing a microsilica addition, compared to control samples.

Lower compressive strength loss following 150 and 300 F-T cycles in the concretes with nanoadditives was also observed by Behfarnia et al. [74]. The study showed 73–83% reductions in the compressive strength loss after 150 F-T cycles in the concretes containing the SiO_2_ additive and (78–80)% reductions in compressive strength loss for the specimens with nano-Al_2_O_3_, compared to control specimens. Furthermore, in the concretes without nanoadditives, an 84% weight loss was observed after 300 F-T cycles, while the samples with nano-SiO_2_ and nano-Al_2_O_3_ showed a weight loss amounting to (4–18)%.

In turn, Quercia et al. [75] reported that the nanosilica addition improved all durability indices, including frost resistance. By analyzing the microstructure, the formation of a dense, homogeneous internal structure of concrete was observed due to the addition of nanosilica.

Recent studies [76,77,78,79] demonstrate the positive effect of nanomaterials on the resistance of concrete to salt crystallization. Nanosilica can improve the resistance of concrete to sulfate attack by using its densifying effect on the microstructure, which slows down the penetration of sulfate ions and water into the concrete.

A study by Ghafoori and Najimi [78] showed that the addition of a nanosilica additive to concrete reduces the cement mortar expansion resulting from sulfate attack. Arel and Thomas [79] found that not only the pozzolanic nature of nanosilica, but also the effect of the nanofiller is responsible for reducing the expansion of cement mortars in a sulfate environment. As a result, a tight mortar microstructure with low porosity is formed after the application of nanofiller.

According to Du et at. [80], the nanomaterial can improve both frost and salt crystallization resistances as a result of the thickening properties of the microstructure, which ultimately leads to hindering the sulfate ions as well as water penetration into the concrete.

Table 7 shows the thermal properties of the concretes.

The thermal conductivity coefficient λ describes the amount of energy that flows through a 1 m-thick layer of material when the temperature difference characterizing both two sides of the material layer tested is 1 K (1 °C). Among others, the lambda value is dependent upon the material porosity and density. The lower the material density and the higher the porosity, the lower the lambda value; this means that the material has greater thermal insulation properties.

The highest λ coefficient was recorded in the concretes containing water with micro-nano ozone bubbles, while the lowest value was observed in reference concretes. In this case, the λ coefficient was 8% lower than that with O_3_. Hence, the concretes with micro-nano gas bubbles produced a denser and less porous structure, in comparison to the control concrete.

Kim et al. [81] observed that using water containing hydrogen nanobubbles in the production of cement mortars reduced thermal conductivity by 30%, compared to tap water.

Saleh et al. [82] observed that the nano-SiO_2_ addition positively affects the heat insulation ability. The study indicated that the thermal conductivity characterizing the concrete with nanoadditive reached a value within the range of (0.5–0.56) W/m °C, while the reference concrete had a thermal conductivity of (1.22–1.34) W/m °C. The authors of [82] also observed a decrease in thermal conductivity along with an increase in the nano-SiO_2_ amount. Saleh cites the small air space formation as the primary reason for the reduced thermal conductivity, which hinders heat transfer.

**Figure 12:**
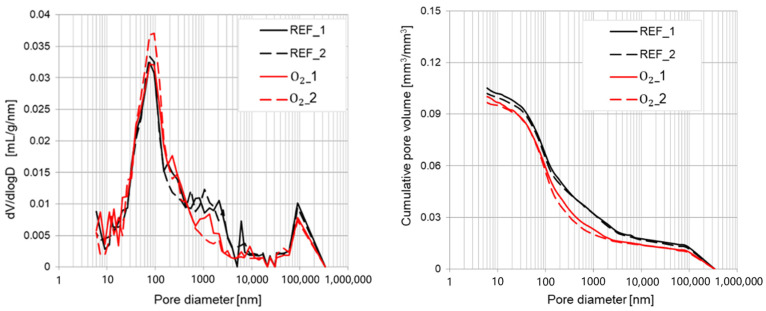
Pore size distribution of the concrete specimens with water comprising micro-nano oxygen bubbles (O_2_) compared to the reference samples (REFs).

**Figure 13:**
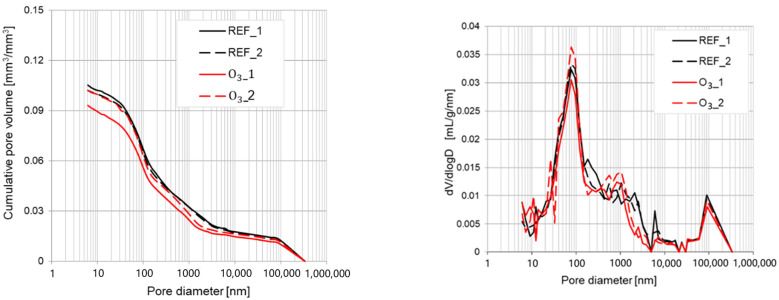
Pore size distribution of the concrete specimens with water comprising micro-nano oxygen bubbles (O_3_) compared to the reference samples (REF).

The influence of tap water with micro-nano oxygen bubbles and micro-nano ozone bubbles exerted on the concrete pore structure was studied. The differential pore volume distributions (dV/dlogD) and the cumulative pore volume results obtained via mercury intrusion porosimetry analysis are shown in Figure 12 as well as Figure 13. The specific parameters of the microstructure of each type of cement paste are presented in Table 8.

The results given in Table 8 are the average value taken over two samples of each type of tested material. The results obtained using mixing water with micro-nano bubbles of oxygen (O_2_) or ozone (O_3_) clearly indicate that it did not significantly affect the pore size distribution of the analyzed cement-based material according to the reference sample with tap water (REF). Each of the tested specimens is characterized by a larger peak in the case of pores with a diameter of about 100 and 100,000 nm. Some differences between analyzed materials might be observed in the case of the structure parameter shown in Table 8.

Small increases were observed in the case of total pore area from 2.50 m^2^/g for the reference sample to 2.57 m^2^/g in the case of O_2_ and 2.60 m^2^/g in the case of the O_3_ specimen and an insignificant change in the case of apparent density. The most significant modifications in the microstructure of analyzed cement pastes were observed for average pore diameter and porosity. Both O_2_ and O_3_ specimens showed a decrease in the average pore diameter and porosity. There were no considerable differences between O_2_ and O_3_ cement pastes in terms of the two parameters mentioned above. The use of liquid nanomaterials, meaning as mixing water with micro-nano bubbles, is quite a novel approach in the recent scientific research [33,36,83,84]. Most of the scientists used nanomaterials in the solid form [85,86,87] or colloids [40,87,88,89,90,91]. In a paper where the impact of hydrogen micro-nano bubbles on the microstructure of cement-based material were investigated (e.g., [34]), the authors observed a significant change in pore size distribution, which did not occur in the case of the presented results for micro-nano oxygen and ozone bubbles.

**Figure 14:**
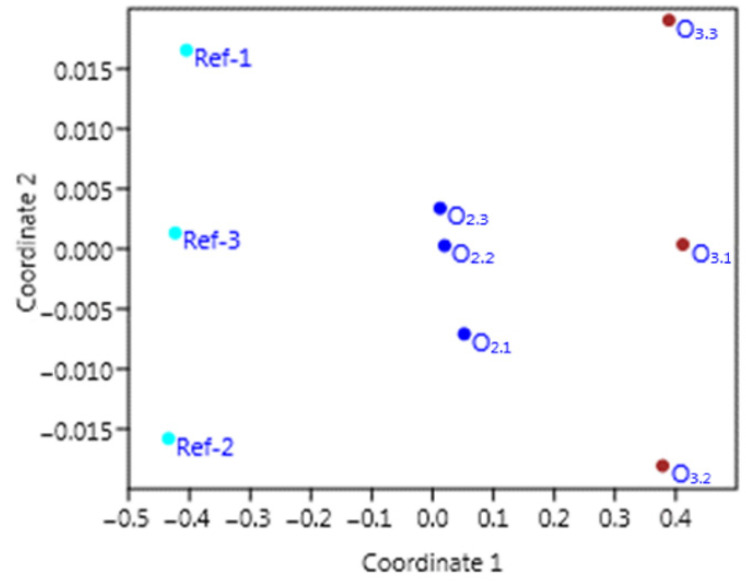
Multidimensional scaling results of effects of adding the water with O_2_ and O_3_ micro-nanobubbles on concrete properties.

A visualization of the similarity levels is shown in Figure 14, prepared on the basis of the multidimensional scaling method (MDS) for the matrix containing all the results of the measurements related to the concrete properties, including water containing O_2_ or O_3_ micro-nano bubbles and reference concretes.

Figure 14 presents the high similarity characterizing the samples of reference concrete in terms of a similar location according to coordinate 1. The group of points showing the properties of the mortars comprising O_2_ M-NB water was located quite far away from the abovementioned reference samples, showing the properties and their overall variation in the reference concrete. The group of points indicating the properties of the concrete containing O_2_ M-NB also had smaller inter-point distances than the other groups of points (Ref and O_3_), showing lesser variability in the results obtained. Another relationship graphically visualized using multidimensional scaling shows that the measurements performed for the O_2_-containing concrete yielded results characterized by greater similarity (lesser distance) to the O_3_ M-NB-water-containing concrete than to the reference sample. In the two-dimensional space, the points that corresponded to the measurement results of the concrete containing O_3_ were furthest from the points, showing the results obtained for the reference mortar.

**Figure 15:**
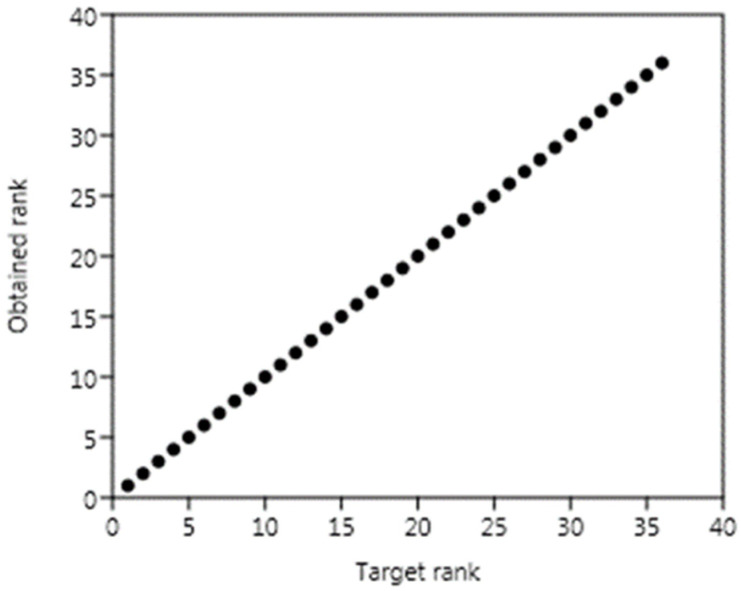
Visualization of the relationships between target rank and obtained rank at STRESS = 0.01 [-].

The MDS analysis also indicates that the STRESS parameter value was very low, i.e., 0.01. This reflects the high goodness of fit between the matrix of the estimated as well as observed distances. Moreover, an almost straight line for the point is shown in Figure 15, along the plane marked as the target rank (X-axis) and the obtained rank (Y-axis), supporting this finding.

The generation of micro-nano bubbles by means of ceramic membranes constitutes one of the numerous available methods. It is also possible to generate micro-nano bubbles via the vortex flow method. In this method, liquid and the gas are forced into a high speed vortex motion, and shear force causes the generation of micro-nano bubbles. Fan et al. [92] successfully generated micro-nano bubbles with an average diameter under 50 nm by means of a venturi [92] as well as gas fed into a pipe. Ahmadi and Darban [93] managed to generate nanoparticles with an average diameter amounting to 130–545 nm by means of a venturi and the hydrodynamic cavitation phenomenon. Wu et al. [94] were able to generate micro-nano bubbles with a diameter of under 500 nm using cavitation induced by highly intense liquid mixing. Another method is acoustic cavitation. Sound waves at a frequency of 20 kHz are generated by an acoustic probe, creating vibrations and transmitting them to a gas nozzle immersed in the liquid. Oeffinger and Wheatley employed this method, introducing a gas jet under constant pressure into the solution, and produced micro-nano bubbles with an average diameter of 400–700 nm [95]. The literature also confirms that frequencies below 20 kHz are harmless to micro-nano bubbles and even allow the generation of additional micro-nano bubbles by cavitating the dissolved gases in the liquid [96]. The application of straightforward, scalable methods to generate micro-nano bubbles characterized by low consumption of energy is crucial for future applications. The obtained knowledge was confirmed via the studies conducted by independent researchers who employed a Nano Sight instrument manufactured by Malvern [97].

**Figure 16:**
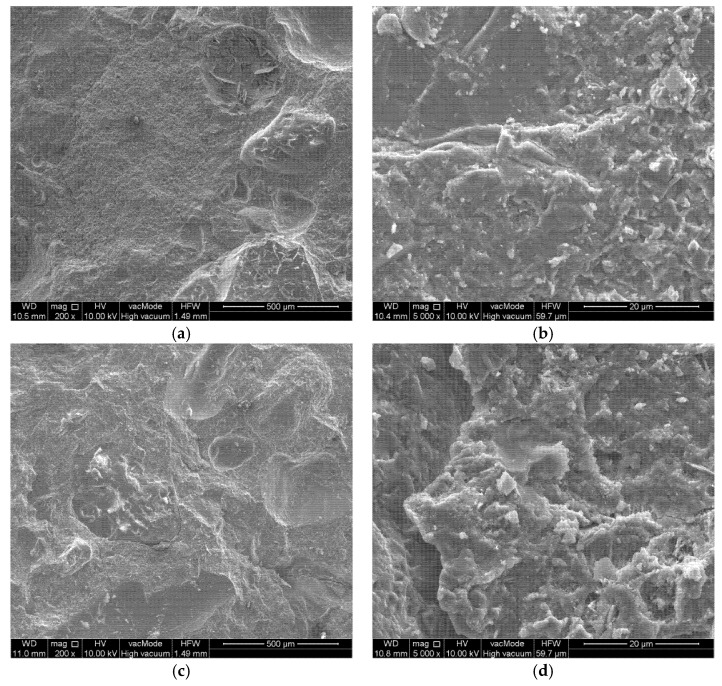

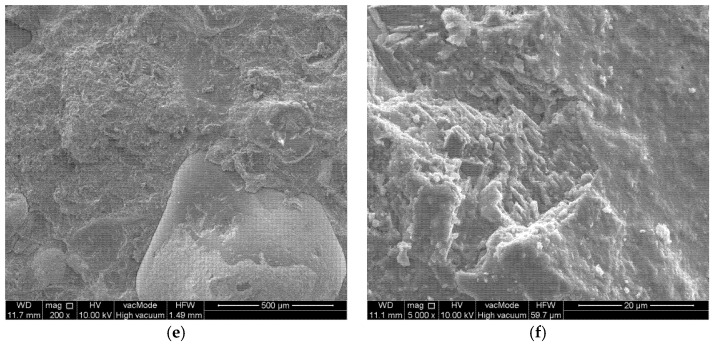
Microstructure of concretes: (**a**) reference—200×, (**b**) reference—5000×, (**c**) concrete with M-NOB—200×, (**d**) concrete with M-NOB—5000×, (**e**) concrete with M-NOzB—200×, (**f**) concrete with M-NOzB—5000×.

The microstructure of studied concretes is shown in Figure 16.

For all concretes in Figure 16a,c,e, a very good adhesion of the cement paste to coarse aggregate was observed. There were no scratches, cracks or weakening of the interfacial transition zone (ITZ) between the aggregate and the cement paste. The bond between the cement paste and aggregate affected the mechanical strength of the concrete (Figure 6 and Figure 7). The structure of the concretes was compact; moreover, no cracks or micro-cracks were visible. When analyzing Figure 16b,d,f of the concretes at 5000× magnification, virtually no differences were observed in the microstructure of the concretes. The content of M-NOBs and M-NOzBs did not increase the porosity of the concrete, and no fine pores were observed in the structure. Conversely, the content of ozone and oxygen resulted in the sealing of the structure. The microstructural observations confirm the results of the porosimetric tests shown in Figure 2 and Figure 3 as well as Table 8.

Feng et al. [98], in their study, showed that the sample containing MNB caused greater homogeneity between the concrete samples and the ITZ, which improved the permeability properties of the concrete. MNB increases the probability of collision between air bubbles and cement and promotes the cement hydration reaction. Calcium silicate hydrate (C-S-H) and hydration products develop more densely, as demonstrated by Kim et al. [65]. As shown in the microstructural studies of [99], the density of cement using MNB batch water is much higher, and the amount of AFt precipitation on the surface of the C-S-H gel is higher, improving early strength.

## 4. Conclusions

The influence of water comprising micro-nano oxygen and ozone bubbles on the mechanical and durability properties of concretes was investigated in this study. The conclusions that can be drawn from the research are as follows:

- Using micro-nano gas bubbles instead of tap water increases the mechanical strength of the produced concretes. The highest compressive strength following 28 days of maturation was observed in the concretes with M-NOzBs, which showed 61% higher strength in comparison with the reference concrete. The study also showed a more rapid strength enhancement between 14 and 28 days of maturation in the samples with micro-nano ozone bubbles.
- The concretes with M-NOzBs and MNOBs obtained 52.3% and 42.7% lower water absorption coefficients, respectively, than the reference concrete. All concretes with micro-nano bubbles showed a decrease in total porosity of about 6.0–7.0% compared to the reference concrete.
- Micro-nano bubble water increases the concrete durability against the damaging effects of frost and salt crystallization. A 57% decrease in compressive strength loss following 150 freezing and thawing cycles was noted in the concretes with M-NOBs, and a 58% reduction in weight loss was seen in the M-NOzB concretes compared to the reference samples.
- This study showed that the concretes containing micro-nano bubbles of oxygen and ozone were characterized by higher thermal conductivity coefficients, λ, compared to the reference concrete. The highest λ coefficient was found in the concretes with micro-nano ozone bubbles.
- The analysis performed using the MDS method, simultaneously considering the results of all analyzed parameters, indicated that the concretes prepared using the water containing O_2_ and O_3_ micro-nano bubbles may be clearly distinguished from the reference samples. The changes corresponding to concrete properties were most evident when the water with O_3_ micro-nano bubbles was added.

Micro-nano bubbles from O_2_ and O_3_ in tap water were found to be highly effective. They can be widely employed for protecting new concrete exposed to aggressive environmental influences, e.g., frost and de-icing salts. An important result of the conducted research is a significant compressive strength enhancement of concrete. The technologies involved in manufacturing high-strength, frost-resistant and salt-resistant concrete for general use should be less expensive to improve large-scale productivity. Micro-nano bubbles from O_2_ and O_3_ can be a cheaper alternative to expensive, chemical anti-frost admixtures.

## Figures and Tables

**Table 1:** Composition of concrete mixtures (kg/m^3^; L/m^3^).

Component	REF	O_2_	O_3_
CEM I 42.5R	373.9	373.9	373.9
Sand 0.0–2.0 mm	914.8	914.8	914.8
Gravel 2.0–8.0 mm	1053.5	1053.5	1053.5
Water	130.9	-	-
Water with micro-nano oxygen	-	130.9	-
Water with micro-nano ozone	-	-	130.9
Hydrophobizing admixture	2.8	2.8	2.8
Superplasticizer	1.9	1.9	1.9

**Table 2:** The chemical composition of CEM I 42.5 R [40].

Compound	Unit	Mass
CaO	(%)	64.41
SiO_2_	(%)	20.23
Al_2_O_3_	(%)	3.62
Fe_2_O_3_	(%)	4.36
MgO	(%)	1.36
Na_2_O	(%)	0.26
K_2_O	(%)	0.5
Na_2_O_eq_	(%)	0.63

**Table 3:** Properties of CEM I 42.5R [41].

Parameters	Unit	Volume
Specific Surface	(cm^2^·g^−1^)	3426
Initial Setting Time	(min)	146
End Setting Time	(min)	190
Specific Gravity	(g·cm^−3^)	3.09
Water Demand	(%)	27.6
Compressive Strength After 2 Days	(MPa)	28.8
Compressive Strength After 28 Days	(MPa)	54.1

**Table 4:** Physical properties of the tested concretes.

	Absorptivity	Bulk Density	Specific Density	Total Porosity	Open Porosity	Water Absorption Coefficient
	(%)	(g/cm^3^)	(g/cm^3^)	(%)	(%)	(g/m^2^·s^0,5^) after 24 h
REF	4.60	2.24	2.59	13.19	10.47	2.812
O_2_	4.39	2.28	2.61	12.73	9.87	1.610
O_3_	4.28	2.27	2.60	12.73	9.76	1.327

**Table 5:** Mechanical properties of the tested concretes.

		REF	O_2_	O_3_
f_cm,14_	(MPa)	25.3	29.4	33.4
SD *		3.45	8.15	12.63
CV **		27.00	27.70	37.82
f_cm,28_	(MPa)	30.8	32.3	49.7
SD *		2.13	5.19	1.59
CV **		10.23	16.08	3.19
f_fm, 28_	(MPa)	4.9	4.9	5.2
SD *		0.25	0.30	0.67
CV **		4.97	6.72	12.82

* SD—standard deviation. ** CV—coefficient of variation, (%).

**Table 6:** Durability properties of the tested concretes.

	Frost Resistance—Loss in Weight	Frost Resistance—Loss in Compressive Strength	Salt Crystallization (+/−) Weight Difference
	(%)	(%)	(%)
REF	0.43	18.49	2.37
O_2_	0.19	8.03	2.21
O_3_	0.02	7.80	2.11

**Table 7:** Thermal conductivity coefficient of concretes.

Series	Units	REF	O_2_	O_3_
λ	(W/m·K)	2.131	2.145	2.302

**Table 8:** Parameters of the microstructure of the cement paste determined by MIP.

	Units	REF	O_2_	O_3_
Total pore area	(m^2^/g)	2.50	2.58	2.60
Average pore diameter	(nm)	72.55	66.45	64.80
Apparent density	(g/cm^3^)	2.55	2.56	2.57
Porosity	(%)	10.36	9.84	9.76

## Data Availability

Not applicable.
